# Single-cell RNA-seq analysis of mouse preimplantation embryos by third-generation sequencing

**DOI:** 10.1371/journal.pbio.3001017

**Published:** 2020-12-30

**Authors:** Xiaoying Fan, Dong Tang, Yuhan Liao, Pidong Li, Yu Zhang, Minxia Wang, Fan Liang, Xiao Wang, Yun Gao, Lu Wen, Depeng Wang, Yang Wang, Fuchou Tang

**Affiliations:** 1 Beijing Advanced Innovation Center for Genomics, Biomedical Pioneering Innovation Center, School of Life Sciences, Peking University, Beijing, China; 2 Bioland Laboratory (Guangzhou Regenerative Medicine and Health Guangdong Laboratory), Guangzhou, China; 3 GrandOmics Biosciences, Beijing, China; 4 Ministry of Education Key Laboratory of Cell Proliferation and Differentiation, Beijing, China; 5 Peking-Tsinghua Center for Life Sciences, Academy for Advanced Interdisciplinary Studies, Peking University, Beijing, China; IMBA, AUSTRIA

## Abstract

The development of next generation sequencing (NGS) platform-based single-cell RNA sequencing (scRNA-seq) techniques has tremendously changed biological researches, while there are still many questions that cannot be addressed by them due to their short read lengths. We developed a novel scRNA-seq technology based on third-generation sequencing (TGS) platform (single-cell amplification and sequencing of full-length RNAs by Nanopore platform, SCAN-seq). SCAN-seq exhibited high sensitivity and accuracy comparable to NGS platform-based scRNA-seq methods. Moreover, we captured thousands of unannotated transcripts of diverse types, with high verification rate by reverse transcription PCR (RT-PCR)–coupled Sanger sequencing in mouse embryonic stem cells (mESCs). Then, we used SCAN-seq to analyze the mouse preimplantation embryos. We could clearly distinguish cells at different developmental stages, and a total of 27,250 unannotated transcripts from 9,338 genes were identified, with many of which showed developmental stage-specific expression patterns. Finally, we showed that SCAN-seq exhibited high accuracy on determining allele-specific gene expression patterns within an individual cell. SCAN-seq makes a major breakthrough for single-cell transcriptome analysis field.

## Abstract 2

This study describes a novel single-cell RNA-seq technology called SCAN-seq which can capture the full-length transcripts in single cells based on the third-generation Nanopore sequencing platform, and demonstrates its performance on mouse preimplantation embryos.

## Introduction

The development of next generation sequencing (NGS) platform-based single-cell RNA sequencing (scRNA-seq) techniques has made great advances during the past decade, and these techniques have accelerated researches in many biological fields. It helped to overcome the challenges in studying rare biological materials and illustrated the heterogeneity within a biological sample [1–2]. The highly parallel scRNA-seq methods such as Drop-seq [3–4] and Microwell-seq [5] have made it feasible to analyze human cell atlas (HCA). However, they relied on NGS platform with short read length (100 to 250 bp). Alternative splicing of transcripts is prevalent in mammalian cells and could make major differences for maintenance of cell identity and function [6–7], many of which could not be detected by NGS platform-based single-cell RNA-seq methods due to their short read length. Therefore, we need new solutions on accurately reporting the complicated alternative splicing events at single-cell resolution.

The third-generation sequencing (TGS) platform has overcome the drawbacks of short read length of NGS and has been applied in direct RNA sequencing [8–9]. However, all the TGS sequencing strategies need large amount of starting materials for library construction, which is difficult to be amplified from an individual cell. Here, we reported a novel method called single-cell amplification and sequencing of full-length RNAs by Nanopore platform (SCAN-seq), which could detect the full-length transcripts in single cells with high accuracy. By pooling amplified full-length cDNAs of 48 individual cells labeled by different Nanopore platform compatible barcodes, the final total cDNA amount was enough for Nanopore library construction and sequencing. The single-cell information could be separated according to the 24-nucleotide (nt) Nanopore platform compatible cell barcodes efficiently. We tested this method in mouse embryonic stem cells (mESCs), and TGS-based SCAN-seq showed comparable sensitivity and higher reliability compared to those NGS-based scRNA-seq methods. Moreover, we directly captured the exact quantity of full-length cDNAs in mESCs, including unannotated ones which could be validated by reverse transcription PCR (RT-PCR)–coupled Sanger sequencing. We further applied this method to investigate the transcriptome in mouse oocytes and preimplantation embryos. Cells at different developmental stages could be clearly distinguished, with each stage of samples well matched the ones analyzed using NGS method. We identified 27,250 unannotated transcripts from 9,338 genes in the mouse preimplantation embryos, and 5 out of 7 candidate unannotated transcripts we chose could be validated by RT-PCR–coupled Sanger sequencing. Finally, we showed that SCAN-seq could accurately evaluate allele-specific transcripts within an individual cell during mouse preimplantation development. Taken together, by taking advantages of both single-cell full-length cDNA amplification and TGS, SCAN-seq showed unique advantages in single-cell transcriptome analysis.

## Results

### Characterization of SCAN-seq technique

**Fig 1:**
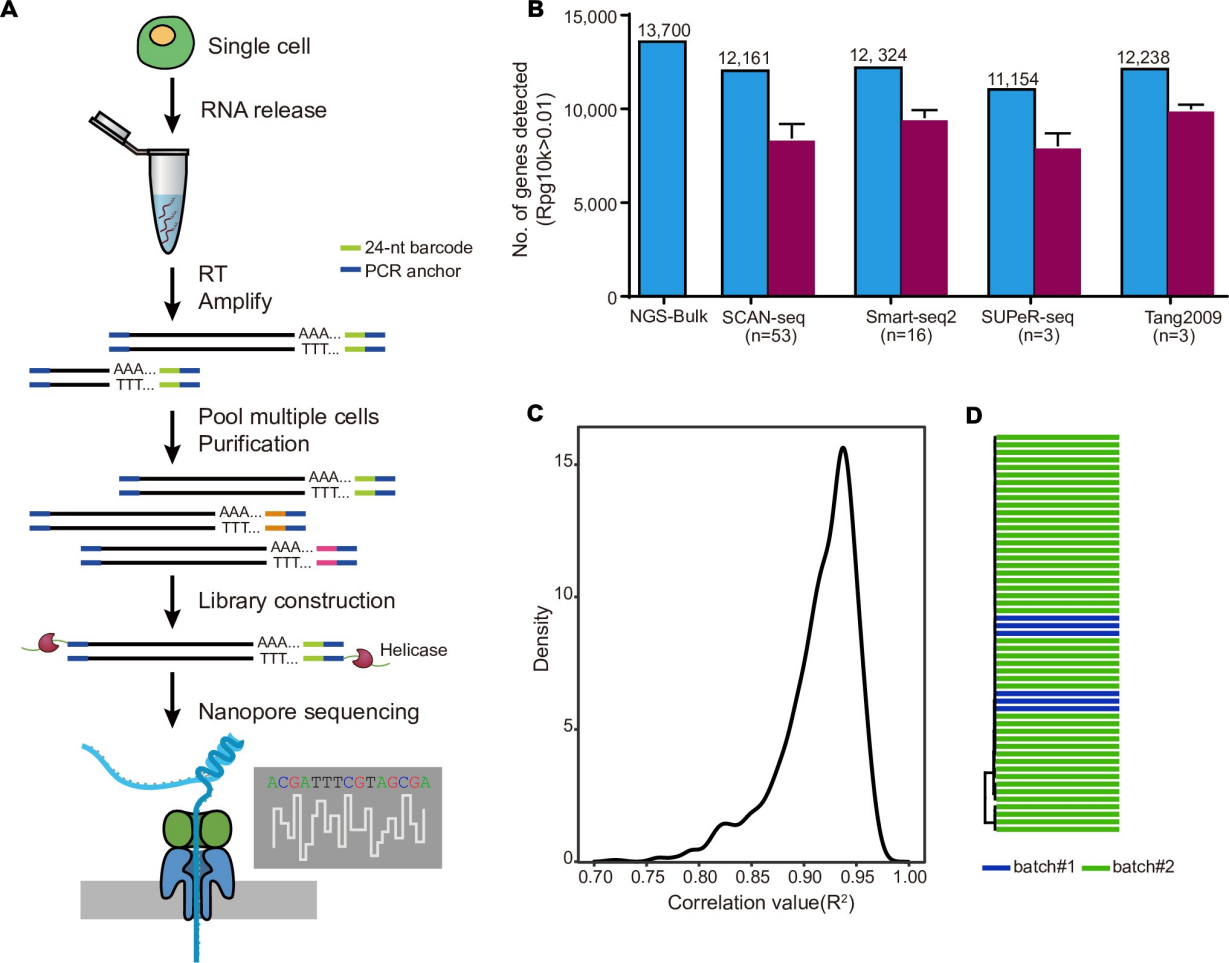
Characterization and evaluation of SCAN-seq. (A) The schematic of SCAN-seq. Briefly, a single cell is lysed to release mRNAs and, the first-strand cDNAs are synthesized using a 24-nt barcode RT primer. After amplification, cDNAs of up to 48 cells are pooled and purified. Then, the full-length cDNAs are used for Nanopore library construction and sequencing. (B) Comparison between the numbers of genes detected by different methods. Both gene number of the group (detected in at least 10% of the cells in the group) and single cells (the purple bars) are shown. The center represents the mean, whereas the error bars represent the SEM. (C) Density plot showing the distribution of global gene expression correlations (Pearson) between every 2 mESCs. Most of the cells showed correlation of over 0.94. (B, C) The numerical data are listed in S1 Data. (D) Dendrogram of mESCs from 2 different experimental batches shows mixed pattern. NGS, next generation sequencing; nt, nucleotide; RT, reverse transcription; SCAN-seq, single-cell amplification and sequencing of full-length RNAs by Nanopore platform; SEM, standard error of the mean.

Current single-cell RNA amplification methods could achieve up to tens of nanogram of cDNAs, which is enough for NGS library construction. While in TGS, near micrograms of starting material were needed for library construction. To develop SCAN-seq, we took advantages of the TGS platform compatible barcoding strategy, through which we could get enough cDNA products by pooling cells with different barcodes. We introduced the official 24-nt barcode sequences of Nanopore in the reverse transcription (RT) primers to avoid cell splitting bias in the TGS data generation. We followed the previous barcoded Smart-seq2 amplification procedure with minor modifications [10–12] and pooled the amplified cDNAs of up to 48 cells in a library (Fig 1A). As the PCR amplification generally show bias on shorter cDNAs, we purified the pooled amplicons twice with 0.6 volume of Ampure beads to remove shortest cDNA products, which are usually primer dimers and cDNAs of partially degraded mRNAs. Then, the purified full-length cDNAs were used for Nanopore library construction and sequencing (Fig 1A). Further gene expression and unannotated transcript analysis were carried out with TGS data in each individual cell (S1A Fig).

We first tested SCAN-seq in mESCs. The length of cDNA products before library construction enriched for fragments of 1,200 to 2,000 bp (S1B Fig). Almost all the full-length reads were longer than 300 bp (S1C Fig), and the length distribution of the reads increased after data collapse as the short reads contained higher ratios of PCR duplicates (S1D Fig). The average ratio of reads mapping for SCAN-seq was 0.87, higher than the standard NGS platform-based scRNA-seq methods. The number of genes and transcripts detected in each cell increased with more reads, while the degree of increases rapidly reduced when the reads went over 100,000 (S2A Fig); thus, we set this number as 1 criterion for filtering cells for downstream analyses. By further requiring the mapping ratio over 85% and number of detected genes more than 3,000, 53 out of 55 mESCs from 2 batches of experiments were kept for further analyses. On average, SCAN-seq could detect over 8,000 genes and 10,000 splice isoforms in each individual mESC (S2A Fig), which was comparable, or even better than those of NGS platform-based single-cell RNA-seq techniques [13] (Fig 1B). As there were 13,700 genes detected in NGS bulk sample, the 12,161 genes obtained by SCAN-seq in mESCs (by requiring detected in at least 10% of the single-cell samples) could detect nearly 90% genes in bulk, representing high sensitivity.

We further evaluated the reproducibility of SCAN-seq by calculating the correlations on global gene expressions between each pair of individual mESCs. The averaged correlation value (R^2^) was 0.9 (Fig 1C and S2B Fig), which was even higher than that of many previous NGS platform-based single-cell RNA-seq methods [13], indicating high reproducibility of SCAN-seq. This could be also revealed by well-mixed pattern of mESCs from 2 different batches in clustering (Fig 1D). Then, we calculated the coverage along the whole transcripts by SCAN-seq, and it showed relatively mild decrease of the coverage from the 3′ to the 5′ end of mRNAs (S2C Fig). This allowed us to get comprehensive information of full-length mRNAs.

**Fig 2:**
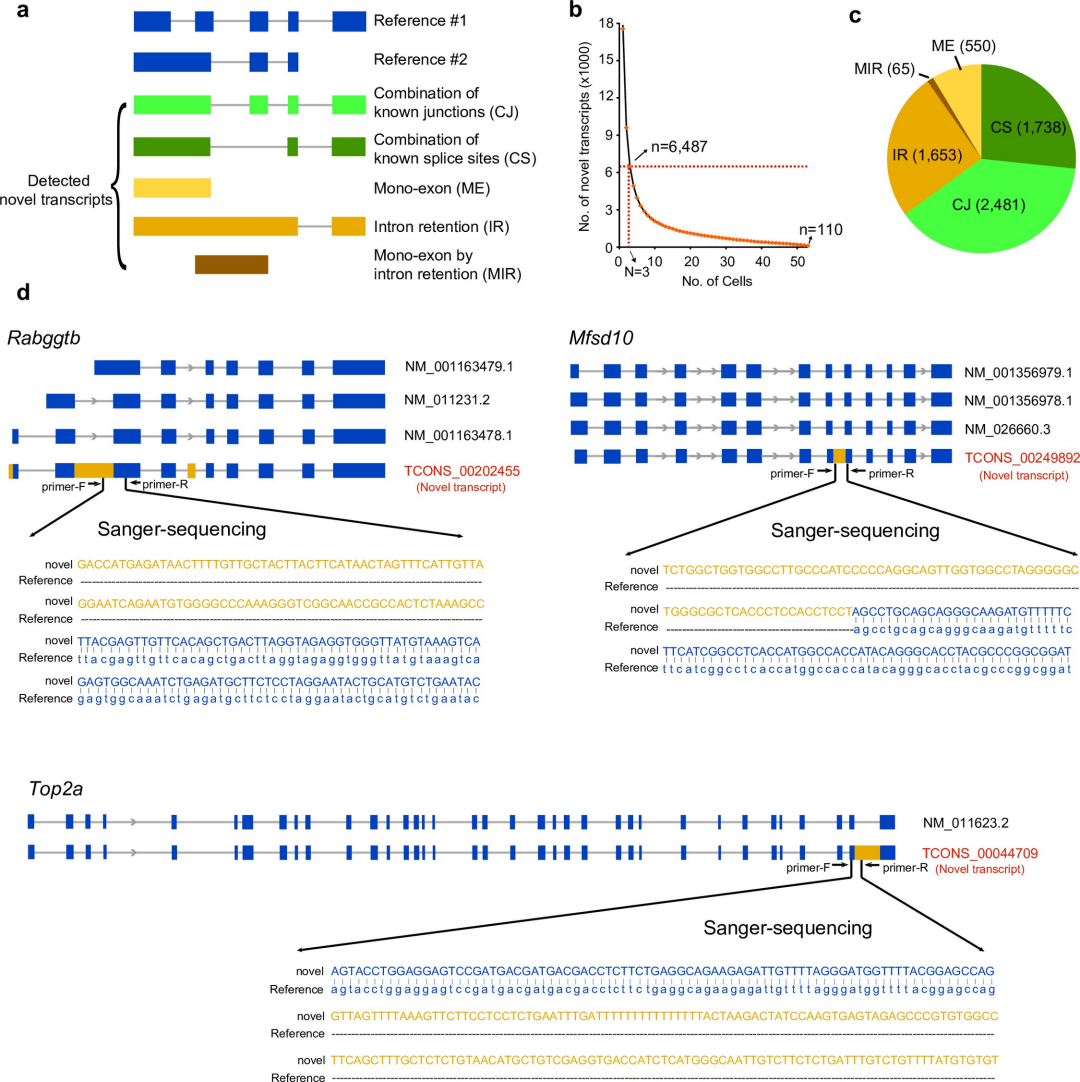
Unannotated transcripts in mESCs. (A) The schematic showing the 5 types of unannotated transcripts. (B) The number of unannotated transcripts detected within different numbers of cells. The unannotated ones captured in at least 3 cells were remained, and we analyzed 110 unannotated transcripts detected in all 53 mESCs. The numerical data are listed in S1 Data. (C) Proportions of these 5 types of unannotated transcripts in mESCs. (D) Examples of unannotated transcripts identified in mESCs. TCONS_00202455 corresponds to a part of the Gene *Rabggtb* sequence. A complete intron between the second and third exon, 25 bp of intron right next to 5′ of the first exon, and 98 bp of intron between the fourth and fifth exon are retained in the RNA. Gene *Mfsd10* has 3 annotated transcripts, and TCONS_00249892 contains the intron between the eighth and ninth exon of the known transcripts. TCONS_00044709 matches partial Gene *Top2a* sequence and contains a complete intron between the 35th and 36th exon of the known transcript. CJ, combination of known junctions; CS, combination of known splice sites; IR, intron retention; ME, mono-exon; mESC, mouse embryonic stem cell; MIR, mono-exon by intron retention.

Next, we referred to an existing algorithm for identifying unannotated transcripts with SCAN-seq data [14]. We searched 5 types of unannotated transcripts: (1) combination of known junctions (CJ), indicating new combinations of previous annotated splice junctions from different transcripts of the same gene; (2) combination of known splice sites (CS), which implies new combinations of annotated splice junctions within the same transcript; (3) mono-exon (ME), deriving from 1 annotated exon; (4) intron retention (IR), which is a very prevalent alternative splicing type that 1 or more introns are preserved in the mRNA; and (5) mono-exon by intron retention (MIR), appearing as 1 or more annotated exons with corresponding flanking introns retained together as a new long exon (Fig 2A). Although we could use assembly to find unannotated transcripts with NGS data, it is difficult to distinguish CJ and CS. Since we were able to get full-length transcripts by SCAN-seq, it was easy to separate these 2 types of unannotated transcripts, indicating higher accuracy of this technique. The number of unannotated transcripts decreased as expected by requiring them to be simultaneously detected in more individual cells. We obtained 6,487 unannotated transcripts, which correspond to 3,834 genes in total by requiring them detected with expression level (RPT10k; see method) over 0.1 and accounted for over 5% of all transcripts of the host genes in at least 3 individual cells. A total of 110 unannotated transcripts were detected in all 53 mESCs analyzed (Fig 2B). Of all these unannotated transcripts, CJ took part of the largest proportion (2,481 out of 6,487), followed by CS (1,738 out of 6,487). A total of 1,653 transcripts belonged to IR group, and ME and MIR possess 550 and 65 transcripts, respectively (Fig 2C). We selected 5 unannotated transcripts for verification using RT-PCR followed by Sanger sequencing (S2 Table). All of them were consistent with the expected transcripts. We listed some unannotated transcripts of different types as examples (Fig 2D). Specifically, we noticed that *Top2a*, an enzyme that is important for cell cycle, was found with IR between the last 2 exons. The high accuracy for unannotated transcript identification demonstrated that SCAN-seq has made great progress in detecting unannotated transcripts at single-cell level.

### Transcriptome analysis of mouse preimplantation embryos using SCAN-seq

We then applied SCAN-seq to systematically study the transcriptome of mouse oocytes and preimplantation embryos. We isolated single blastomeres of embryos (C57BL/6J♀ × DBA/2C♂) at different developmental stages according to diverse digestive conditions. The inner cell mass (ICM) and trophectoderm (TE) cells in blastocysts were briefly predicted through detecting the abundance of *Nanog* and *Cdx2* in the amplified cDNAs by fluorescent quantitative PCR. The confirmation of cell identity at blastocyst stages were based on clustering of cells according to the expression levels of well-known ICM and TE marker genes (*Nanog*, *Sox2*, *Fgf4*, *Lifr*, *Gata4*, *Gata6*, *Sox17*, and *Foxa2* for ICM and *Cdx2*, *Eomes*, *Elf5*, *Gata3*, and *Tfap2a* for TE). For a total of 213 blastomeres we sequenced, 122 were left after stringent filtering (Table 1). The saturation curve indicated that we could detected a sufficient number of genes and isoforms in individual cells at distinct developmental stages with 100,000 mapped reads, while the abundance of genes and transcript isoforms varied with enough reads (S3 Fig). Oocytes and zygotes were detected with highest number of genes on average (12,360 and 12,467 detected genes, respectively) in each individual cell (S4A Fig). The number of detected genes in each blastomere decreased along the developmental stages, reaching the lowest at 8-cell stage (6,730 on average). This should be caused by the global degradation of maternal RNAs during cell division. The gene content in morular cells increased (8,751 genes on average in an individual cell), indicating a boost of de novo gene transcription at this stage.

**Table 1:** Sample collection of mouse oocytes and preimplantation embryos.

Stage	No. of collected cells	No. of cells after filter	No. of embryos	Genetic background(♀x♂)
MII oocyte	10	7	10	C57BL/6J × DBA/2C
MII oocyte	16	16	16	DBA/2C × C57BL/6J
Zygote	10	9	10	C57BL/6J × DBA/2C
2-cell_early	21	13	11	C57BL/6J × DBA/3C
2-cell_late	20	18	10	C57BL/6J × DBA/4C
4-cell	24	14	9	C57BL/6J × DBA/5C
8-cell	24	12	5	C57BL/6J × DBA/6C
Morula	24	23	3	C57BL/6J × DBA/7C
Blastocyst	80	26	18	C57BL/6J × DBA/8C
Blastocyst	69	49	4	DBA/2C × C57BL/6J

MII, metaphase II.

**Fig 3:**
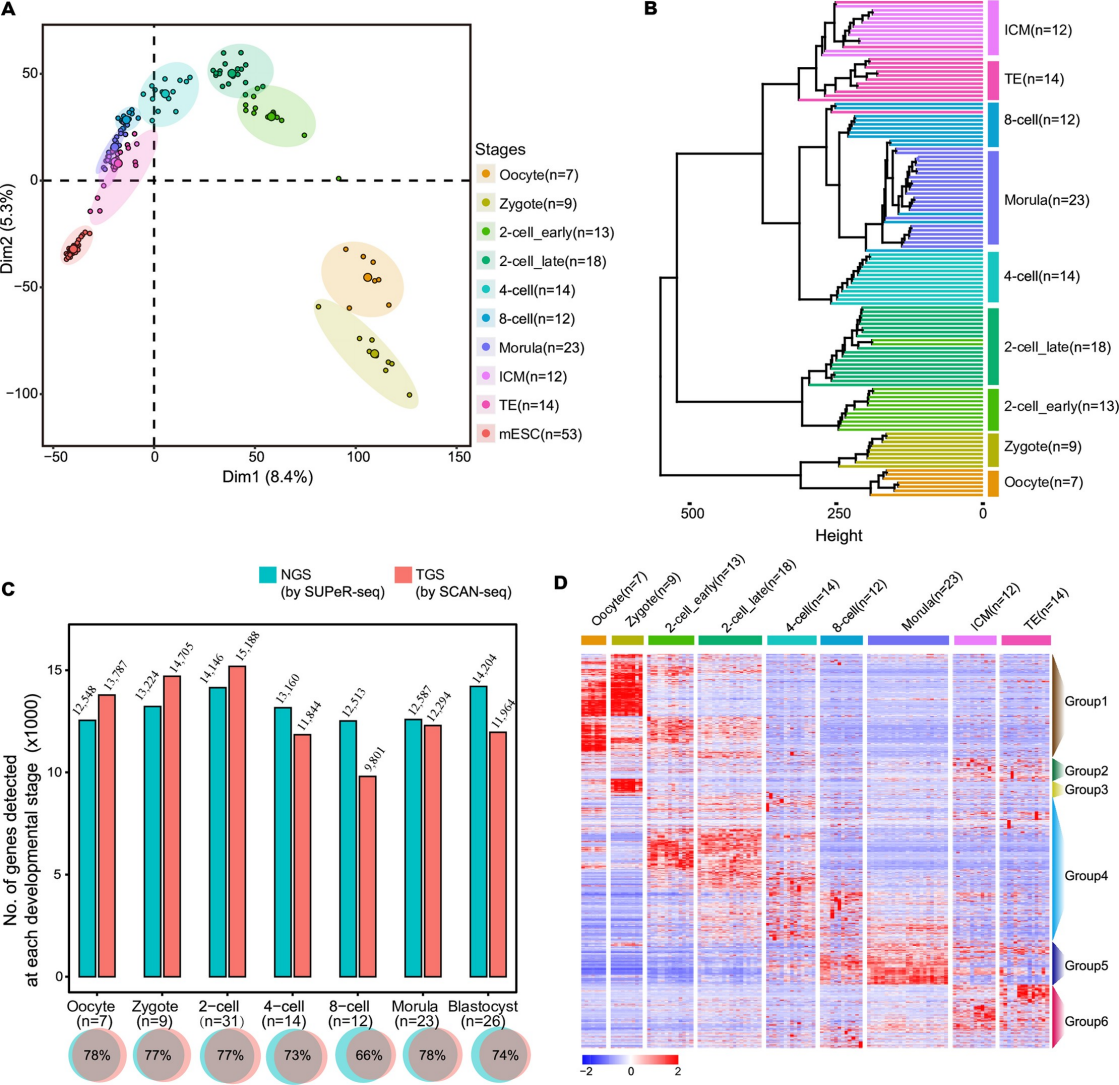
Transcriptome analysis in mouse preimplantation embryos. (A) PCA of mESCs and all blastomeres at different developmental stages. (B) Hierarchical clustering of all blastomeres at different developmental stages. Cells of the same stage clustered together and the adjacent stage showed shorter distances. (C) Comparison between the number of genes detected at each developmental stage by NGS and TGS. SCAN-seq detected more genes before 4-cell stage. And 66% to 78% genes were overlapped by these 2 methods at the same stage. (D) Heatmap of gene expressions showing developmental stage-specific patterns. A total of 2,252 genes were divided into 6 groups according to their expression patterns. (A, C, D) The numerical data are listed in S1 Data. ICM, inner cell mass; mESC, mouse embryonic stem cell; NGS, next generation sequencing; PCA, principal component analysis; TE, trophectoderm; TGS, third-generation sequencing.

We performed principal component analysis (PCA) of all the blastomeres at different developmental stages together with mESCs (Fig 3A). As expected, cells at each developmental stage clustered together on the PCA map and showed a continuous distribution from oocyte, zygote to the ICM, and TE cells at blastocyst stage. The most dramatic transcriptome shift happened at 2-cell stage, exhibiting the largest distance to the previous developmental stages. The global gene expression pattern from morula stage to blastocyst was similar as they could hardly separate on PCA map. Cells at blastocyst stage were more heterogeneous than those at morula stage, indicating more cell fate differentiation at this stage. While the pluripotent mESCs, as the cultured cell line initially derived from ICM cells, were more homogeneous and closer to the blastocyst cells (Fig 3A). Further hierarchical clustering analysis revealed that nearly all cells belong to the same developmental stage could be clustered together, and the adjacent stage of samples showed shorter distances (Fig 3B). We then compared the results to the pairwise data generated by single-cell universal poly(A)-independent RNA sequencing (SUPeR-seq), a representative of scRNA-seq method using NGS platform [15]. SCAN-seq could detect more genes in samples before 4-cell stage. Fewer genes in 4-cell and samples at later stages might be due to the fact that SUPeR-seq used a whole embryo (at least 4 blastomeres) as input for amplification in a reaction, while SCAN-seq used only 1 blastomere (Fig 3C). Nevertheless, both methods showed high consistency on gene detection at each developmental stage, with 66% to 78% overlapped genes (Fig 3C). The stage-specific genes identified using SCAN-seq also matched well with those using SUPeR-seq (S4B Fig), indicating high accuracy and reliability of SCAN-seq data.

To figure out the candidate genes regulating mouse preimplantation development, we searched for differentially expressed genes (DEGs) between every 2 stages of cells and re-clustered all the DEGs according to their expression patterns in samples across all developmental stages (Fig 3D). A total of 2,521 genes could be separated into 6 groups (S1 Table). Group 1 genes such as *Bmp15*, *ZSCAN4−ps1*, *Dyp30*, *Ampd3*, *Tcl1*, and *Clock* showed highest expression levels in oocyte, zygote, and 2-cell stage embryos. These should be maternal genes and gene ontology (GO) analysis showed that they enriched for biological terms related to cell cycle (S4C Fig). The expression of group 2 genes elevated in oocyte, zygote, 2-cell embryos, and ICM cells. The representative genes included *Nanog* and *Lama1*. Genes in group 3 were specifically highly expressed in zygote, such as *Arg2*, *Krt12*, and *Sgcd*, related to regulation of protein and RNA transport. Group 4 genes, including *Egfr*, *Eif4g1*, *Abcf1*, *Kdm5b*, *and Hook1*, were up-regulated during 2-cell to 8-cell stage. These genes enriched for biological terms related to metabolism of RNA, chromatin organization, and cell cycle process. Genes in group 5 showed elevated expression levels from 8-cell stage onwards, and they were strongly associated with GTP hydrolysis and regulation of translation. The representative genes in group 5 included *Rpl27a*, *Gata6*, *Eif5*, etc. We found that the blastocyst specific genes, such as *Pou5f1*, *Src*, *Sox17*, *Aimp1*, and *Cdc37*, in group 6 showed enrichment in cytoskeleton organization, embryonic morphogenesis, cortical cytoskeleton organization, and ectodermal cell differentiation. This indicated that cells at blastocyst stage already began to differentiate with committed cell fate (S4C Fig).

### LncRNAs detected in mouse preimplantation embryos

Long noncoding RNAs (lncRNAs) are important regulators for gene expression regulation. They are also reported to be involved in early embryonic development [16–17]. To investigate the lncRNAs captured by SCAN-seq, we extracted reads mapping to intergenic regions and then mapped the reads to the NONCODEv5 lncRNA database (S4 Table). It is interesting that the intergenic reads were dramatically increased at the 2-cell stage and then decreased along with the developmental stages (S5A Fig). This indicates de novo expression of large abundance of lncRNAs, which were possibly involved in the zygotic gene activation. We further investigated whether lncRNAs play roles in mouse preimplantation development by analyzing the stage-specific lncRNA expressions. For the total of 2,742 lncRNAs we observed in the embryonic samples, 1,032 lncRNAs showed stage-specific expression pattern (S5B Fig), most of which were highly detected before 4-cell stage, indicating potential regulation roles for preimplantation development.

### Unannotated transcripts detected in mouse preimplantation embryos

**Fig 4:**
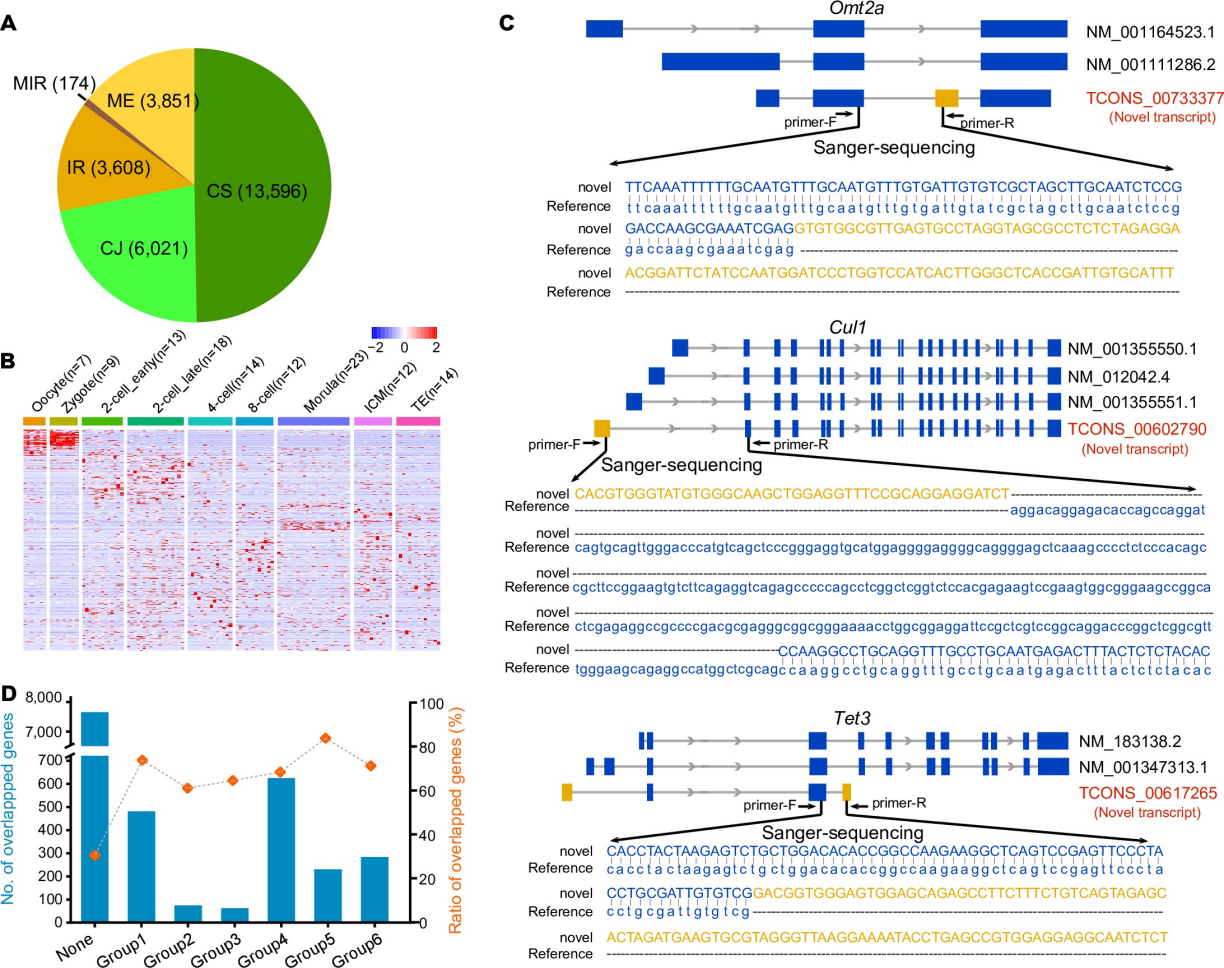
Unannotated transcripts in mouse oocytes and preimplantation embryos. (A) Proportions of these 5 types of unannotated transcripts in mouse oocytes and embryonic cells. (B) Heatmap showing the expression levels of unannotated transcripts in all cells. The numerical data are listed in S1 Data. (C) Examples of unannotated transcripts. TCONS_00733377 matches a part of the Gene *Omt2a* sequence. It contains an extra exon from an intron region. Gene *Cul1* has 3 annotated transcripts, and TCONS_00602790 has a different 5′ UTR. TCONS_00617265 contains 2 annotated exons of *Tet3*, and 2 additional exons came from extra 5′ UTR region and an intron region. These 3 transcripts were verified in mouse oocytes. (D) Comparison between genes generating unannotated transcripts and the 6 groups of stage-specific genes. The histogram showing the number of genes allocating in each group. The ratio of the overlapped genes in each group is also shown. The numerical data are listed in S1 Data. CJ, combination of known junctions; CS, combination of known splice sites; IR, intron retention; ME, mono-exon; MIR, mono-exon by intron retention; UTR, untranslated region.

Likewise, we searched unannotated transcripts in all the preimplantation samples and identified 27,250 unannotated transcripts, which corresponded to 9,338 genes (S2 Table). Different to the ratios in mESCs, CS type took almost half of all unannotated transcripts (13,596 out of 27,250) in mouse embryonic samples (Fig 4A). We then performed hierarchical clustering of these unannotated transcripts according to their expression levels in all embryonic cells. Many of them show developmental stage-specific expression patterns (Fig 4B). We then selected 7 unannotated transcripts which showed higher expression levels in either the oocyte or the blastocyst stage for verification. Five candidates (71%) were consistent with the expected transcripts according to RT-PCR–coupled Sanger sequencing (S2 Table), including protein structure regulation related gene *Cul1* and the epigenetic modification gene *Tet3* (Fig 4C). Then, we wondered how many of the unannotated transcripts were generated by the stage-specific genes. We compared these genes with the 6 groups of genes identified previously. Although 7,574 genes showed no stage-specific expression patterns and only 1,056 genes overlapped with the 6 groups of genes, the stage-specific genes showed higher ratios to be detected with unannotated transcripts (Fig 4D), especially for the maternal genes (group 1) and 8-cell stage onwards expressed genes (group 5) (73% and 83%, respectively). The identification of such a large number of unannotated transcripts provides a valuable resource for further study of their function in the development of mouse preimplantation embryos.

### Allelic-specific and parental-specific expression analysis during mouse preimplantation embryo development

Diploid organisms have 2 alleles of genes in each individual cell, with 1 from mother (maternal allele) and the other from father (paternal allele). A gene is usually expressed from both alleles, which is called biallelic expression, or from only 1 allele, which is called monoallelic expression. Monoallelic gene expression occurs when 1 allele is actively expressed, whereas the other is silent [18–19], and unbalanced allelic gene expression is a significant genetic regulatory mechanism. Parental-specific genes expression is involved in many diseases such as diabetes, inherited diseases, and cancer [20–21]. Therefore, investigating allele-specific gene expression patterns is important, and such evaluations were usually done through NGS technology [22–23].

**Fig 5:**
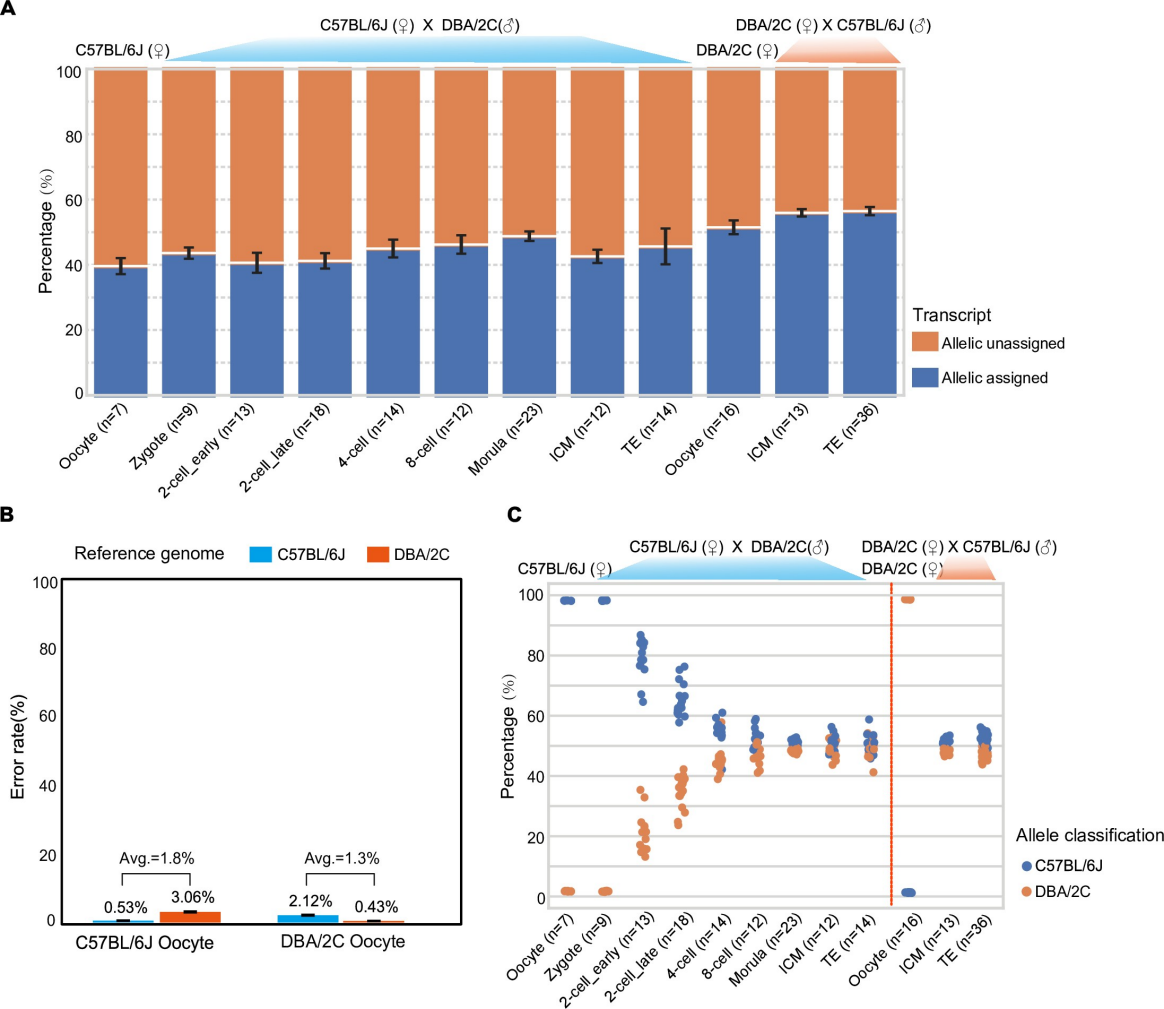
Analysis of allele-specific transcripts. (A) The percentage of allele-specific transcripts at each stage during mouse preimplantation development. (B) Error rates for SNP detection when aligned to C57BL/6J and DBA/2C reference genomes. (C) Percentage of transcripts which can be assigned to each allele in each individual cell. (A–C) The numerical data are listed in S1 Data. ICM, inner cell mass; SNP, single nucleotide polymorphism; TE, trophectoderm.

To check whether we could accurately specify paternal and maternal expressions in an individual cell, we calculated transcripts belonging to C57BL/6J and DBA/2C, respectively, according to different single nucleotide polymorphism (SNP) between these 2 strains in every blastomere of all embryonic stages we analyzed. Of all the expressed genes in each single cell at each developmental stage, 40% to 55% transcripts (correspondingly 45% to 65% of the genes) were detected with at least 1 discriminable SNP (Fig 5A and S6 Fig). We also found that there was a significant bias in calculating maternally expressed genes when using each strain’s reference genome for mapping (Fig 5B). Thus, we mapped the full-length reads to both reference genomes and calculated the paternal and maternal read counts in every individual cell and then averaged the results from both situations (S3 Table). In this way, the averaged error rates for SNP identification were 1.8% for identifying C57BL/6J allelic transcripts and 1.3% for identifying DBA/2C allelic transcripts (Fig 5B). As expected, the ratios of maternal allele expressed genes were near 100% at oocyte and zygote stages and then gradually decreased because of the maternal RNA digestion and zygotic gene activation (Fig 5C). The paternal allelic genes already took about a quarter of all transcripts at early 2-cell stage and nearly 40% at later stage, indicating large scale of gene expression activation at 2-cell stage. This was consistent to the previous observation that genes in group 4 were enriched for genes related to the metabolism of RNA (S4C Fig). Different from the previous results by Deng and colleagues [22] that the transcript amounts from both alleles are almost equal, we found that the total amount of transcripts from the maternal genome was still slightly higher than those from the paternal genome. In our dataset, transcripts from both alleles reached equal abundance at blastocyst stage in both ICM and TE cells. To verify this rigorously, another 16 metaphase II (MII) oocytes of DBA/2C and 49 blastomeres from reciprocally crossed embryos at blastocyst stage were also analyzed (Fig 5C). The results suggested a reliable estimation of allele-specific gene expression by SCAN-seq.

## Discussion

Current NGS platform-based scRNA-seq methods can offer information of expression levels and splicing events for each gene. It is practically impossible to capture the full-length transcripts of longer genes in single cells because of the limitation of short read length of the NGS platform. SCAN-seq took advantages of acquiring full-length cDNA with TGS and makes it implementable at single-cell level. SCAN-seq not only got the results obtained using the current NGS platform-based scRNA-seq methods, but also captured the intact cDNA molecules directly (with the longest cDNA captured in mESCs as 10,050 bp, from the gene *Fasn* with 43 annotated exons), which is convenient and accurate for RNA isoform analysis. Moreover, SCAN-seq could specify the template strand for transcription of a specific RNA molecule, which is practically impossible for the previous NGS platform-based full-length cDNA scRNA-seq methods without specific labeling (S5 Table).

According to the transcriptome analysis of mouse preimplantation embryos using SCAN-seq, we validated that this new method is accurate to distinguish cells at different developmental stages. Additionally, this method can investigate allele-specific gene expression patterns with high accuracy. In summary, SCAN-seq provides a great basis for further study of different biological systems at single-cell resolution.

There are also some limitations for SCAN-seq. For example, the relative high cost of TGS sequencing. The sequencing cost for an individual cell is US$60 in the case of 48 cells as a sequencing library. This made it unaffordable to analyze large number of individual cells. Secondly, due to the relative high error rate of Nanopore sequencing, it is difficult to do absolute counting of transcripts using unique molecular identifiers (UMIs). Therefore, there would be bias in gene expression analysis, caused by the PCR amplification errors. Currently, there is also possible way to evaluate the absolute copy numbers of the transcripts in such TGS based scRNA-seq methods. The most common strategy is to combine NGS data for correction of TGS results [24–26]. In this way, both NGS and TGS libraries need to be constructed with the same sample, increasing the experimental and sequencing costs, and there are fewer TGS reads assigned for downstream analysis. We investigated the published TGS based scRNA-seq methods [24–28] and compared them with SCAN-seq in several aspects (Table 2). SCAN-seq showed clear advantages in cell capture efficiency and gene detection ability by just relying on TGS platform. The throughput of SCAN-seq can also be improved by involving more types of cell barcodes or using combined barcodes on both ends of the transcripts. As for absolute counting of transcripts, 1 applicable way is to use spike-ins, such as External RNA Controls Consortium (ERCC) for copy number evaluation in the future. In summary, SCAN-seq turns out to be a great breakthrough in single-cell transcriptome study using TGS technology, and it will drive research in many biological fields.

**Table 2:** Comparison of SCAN-seq to current TGS base scRNA-seq methods.

Method	Amplification	Sequencing platform	No. of cells in a library	Cell capture efficiency	No. of UMIs per cell	No. of full-length genes per cell	Experiment cost per cell
SCAN-seq	Smart-seq2	Nanopore	10–48	80%	–	8,000	US$3
R2C2 [27–28]	Smart-seq2	Nanopore	1–96	64%	–	532	US$7
ScISOr-Seq [24]	10x Genomics	NGS+Pacbio	>5,000	40%	260	129	US$0.3
ScNaUmi-seq [25]	10x Genomics	NGS+Nanopore	100–1,000	40%	6047	2,427	US$5
RAGE-seq [26]	10x Genomics	NGS+Nanopore	2,500–6,000	18.7%	–	TCR/BCR mRNA	US$0.5

BCR, B cell receptor; NGS, next generation sequencing; RAGE-seq, Repertoire and Gene Expression by Sequencing; R2C2, Rolling Circle Amplification to Concatemeric Consensus; SCAN-seq, single cell amplification and sequencing of full-length RNAs by Nanopore platform; ScISOr-Seq, single-cell isoform RNA-Seq; ScNaUmi-seq, Single-cell Nanopore sequencing with UMIs; scRNA-seq, single-cell RNA sequencing; TCR, T cell receptor; TGS, third-generation sequencing; UMI, unique molecular identifier.

## Methods

### Ethics statement

All animal experiments were performed according to the guidelines of the Institutional Animal Care and the Ethics Committee of the Peking University (Beijing, China). The research license number is LSC-TangFC-4.

### Collection of mouse MII oocytes and preimplantation embryos

The MII oocytes were collected from the C57BL/6J (C57) mice, and preimplantation embryos were collected after the 6- to 8-week-old C57 female mice mated with DBA/2NCrl (DBA) male mice. To induce ovulation, the female mice were injected with 5IU of pregnant mare’s serum gonadotropin (PMSG) (Ningbo SanSheng Biological Technology, Ningbo, Zhejiang, China, Cat. 110044564) and then after 46 to 48 hours 5IU of human chorionic gonadotropin (hCG) (Ningbo SanSheng Biological Technology, Cat. 50030248). The MII oocytes and embryos of each stage during preimplantation development were collected at defined time periods after hCG administration [29]: 20 hours (MII oocyte), 22 to 24 hours (zygote), 30 to 32 hours (early 2-cell), 46 to 48 hours (late 2-cell), 54 to 56 hours (4-cell), 68 to 70 hours (8-cell), 78 to 80 hours (morula), 88 to 90 hours (early blastocyst), and 108 to 116 hours (late blastocyst).

### Single-cell isolation from preimplantation embryos

To isolate single cells from embryos, we first eliminated granulosa cells by putting the MII oocytes and embryos at zygote and 2-cell stages in hyaluronidase (Sigma, St. Louis, Missouri, United States of America, Cat. V900833). Then, we transferred the embryos into the Tyrode’s solution (Sigma, Cat. T1788) to digest the zona pellucida. Next, we used 1:3 dilution of TrypLE (Invitrogen, Carlsbad, California, USA, Cat. 12605010) in Accutase (Invitrogen, Cat. A1110501) to dissociate embryos into single cells. Incubation time varied from 30 seconds to 30 minutes depending on the embryonic stage.

### SCAN-Seq single-cell amplification

After digestion, single cells were placed into 2-μL lysis buffer by mouth pipette. The cell lysis buffer contained 2U RNase Inhibitor (Takara, Beijing, China, Cat. 2313B), 0.0475% Triton X-100 (Sigma-Aldrich, St. Louis, Missouri, USA, Cat. X100), 2.5-μM dNTP mixture (Thermo, Waltham, Massachusetts, USA, Cat. R0193), and 0.75-μM RT primer (AAGCAGTGGTATCAACGCAGAGTAC-XXXXXXXXXXXXXXXXXXXXXXXX-T25, with X representing the nucleotide of cell-specific barcode). We thoroughly vortexed the tubes for 60 seconds and incubated at 72°C for 3 minutes to release the linearized RNA molecules and immediately transferred them on ice. Then, 2.85 μL of RT mixture which comprised of 50U SuperScript II reverse transcriptase (Invitrogen, Cat. 18064071), 5U RNase Inhibitor, 5X Superscript II first-strand buffer, 5M betaine (Sigma-Aldrich, Cat. B0300), 25 mM DTT, 30 mM MgCl2 (Sigma-Aldrich, Cat. 63020), and 1.75-μM TSO primer (AAGCAGTGGTATCAACGCAGAGTACATrGrG+G, rG represents riboguanosines and +G represents the locked nucleic acid (LNA)-modified guanosine) was added into each tube. The RT reaction was carried out at 25°C for 5 minutes, 42°C for 60 minutes, 50°C for 30 minutes, and 70°C for 10 minutes. After that, 7.5-μL PCR mixture that included 2× KAPA HiFi Hot-Start Ready Mix and 300 nM of ISPCR oligo (AAGCAGTGGTATCAACGCAGAGT) was added into each tube. The amplification was performed by the following program: 4 cycles at 98°C for 20 seconds, 65°C for 30 seconds, and 72°C for 5 minutes, followed by 16 cycles at 98°C for 20 seconds, 67°C for 15 seconds, and 72°C for 5 minutes, with a final cycle at 72°C for 5 minutes. Then, we pooled the cDNAs of different cell barcodes together and purified twice with 0.6X Ampure XP beads (Beckman, Brea, California, USA, Cat. A63882). A total of 400 ng to 1 ug cDNA products were used for further library construction.

### SCAN-Seq library preparation and sequencing

We constructed the library for Nanopore sequencing using Ligation Sequencing Kit 1D (ONT, Beijing, China, Cat. SQK-LSK109) following the instructions. Briefly, the cDNA fragments were end-repaired and added dA-tailed using the Ultra II End Prep module (NEB, Ipswich, Massachusetts, USA, Cat. E7546) and then tethered to 1D adapter by using Quick Ligation Module (NEB, Cat. E6056). After that, each cDNA library was loaded into 1 FLOPRO002 flow cell and sequenced on PromethION.Beta.

### Preprocessing of SCAN-seq Data

We used MinKNOW (v3.6.3) and Guppy (v3.1.5) to generate the fastq data from electric signals. After that, we applied nanoplexer (https://github.com/hanyue36/nanoplexer/) to demultiplex barcode for each cell in the library. Specifically, the 150 bp on both ends of the reads were extracted and mapped to cell barcode sequences, obtaining a score for each barcode. The reads were assigned to difference cells based on the highest barcode mapping score, and those with score below 31 were discarded. Then, the low-quality reads (qscore <7) and short reads (length <100 bp) were discarded using nanofilt (v2.5.0) [30]. The left reads were identified, oriented, and trimmed using Pychopper (v2.3) (https://github.com/nanoporetech/pychopper). We obtained the full-length reads from the previous steps according to the PCR anchor sequence (AAGCAGTGGTATCAACGCAGAGTAC) and the cell barcode on both ends of the reads. These full-length reads were aligned to mouse genome and transcriptome (Ensembl, GRCm38.90), respectively, with minimap2 (v2.1) [31].

The genome alignments were performed with the arguments “-ax splice -uf -k14—secondary = no”, and transcriptome alignments used the arguments “-ax map-ont -N 100 -p 0.99.” We filtered the cells with number of full-length reads less than 100,000, bases mapping ratio (to the genome) less than 0.85, or number of detected genes fewer than 3,000.

### Quantitative analysis of transcripts and genes

Based on the number of full-length reads aligned to the known transcriptome, we quantified genes and transcripts using Salmon (v0.14.1) [32] with the parameter as “—noErrorModel -l U.” Then, the expression level was calculated as read counts per 10,000 mapped reads for each gene (RPG10k) and transcript (RPT10k) [33].

### LncRNA detection

The quality passed reads were mapped to the mouse genome, and the mapped reads in the bam file were marked with identities of exon, intron, and intergenic regions. After removing redundancy, the number of reads of each type within each single cell was calculated, and the intergenic reads were mapped to the NONCODEv5 lncRNA database (http://noncode.org/). Quantification of lncRNA expressions was done as the same with the known transcripts.

### Analysis of unannotated transcripts

With the full-length reads mapping to the reference genome in each cell, we performed clustering analysis with pinfish (v0.1) (https://github.com/nanoporetech/pinfish) and filtered out the low-depth read clusters (<2 reads for known exon sites and <5 reads for regions nonoverlapped with known exons). Thus, we obtained mapping intervals with high confidence and used the collapse_isoforms_by_sam.py (https://github.com/Magdoll/cDNA_Cupcake/wiki/Cupcake:-supporting-scripts-for-Iso-Seq-after-clustering-step) script in the cDNA_Cupcake (v8.5) to further remove redundancy. Then, we made comparison between the deduplicated sequences and the mouse reference annotation (Ensemble GRCm38.90) using SQANT2 (v3.8) [14]. Sequences inconsistent with the annotations were selected as unannotated candidate transcripts in each cell, which were further classified as the 5 types in Fig 2A. To search for unannotated transcripts of high confidence, we used gffcompare (v0.10.6) [34] to combine the self-annotated unannotated transcripts in all cells. Then, within each single cell, we calculated the expression level for each novel transcript using Salmon (v0.14.1) with the parameter as “—noErrorModel -l U,” and we defined those with RPT10k less than 0.1 as 0. To remove the artificial novel transcripts as much as possible, we further calculated the ratio of each transcript to the corresponding host gene and removed those accounted for less than 5%. Based on the above criteria, we only retained those simultaneously identified in at least 3 cells as unannotated transcript in each cell type. For unannotated transcripts in the mouse embryos, gffcompare (v0.10.6) was used 1 more time to merge and re-annotate all transcripts at different stages. Meanwhile, the expression levels of unannotated transcripts were calculated together with the known transcripts (Fig 4B).

### Processing of NGS data

The NGS data were downloaded from the GEO database (https://www.ncbi.nlm.nih.gov/geo/query/acc.cgi?acc=GSE53386), and quality control was performed using Fastp (v0.19.6) [35]. Then, we used Salmon (v0.14.1) with the default parameter as “—libType IU” to quantify gene expressions (RPG10k) with the clean data.

### Stage-specific gene expression analysis

The DEGs between each 2 adjacent embryonic stages were calculated using R package DEseq2 [36]. The top 300 DEGs in each group were merged, generating a total list of 2,251 genes. Then, these genes were clustered according to their expression patterns in all stages using pheatmap package in R (Fig 3D) and finally divided into 6 groups.

### PCA and clustering analysis of cells in mouse preimplantation embryos

The PCA analysis was performed using R packages. Firstly, dimension reduction process was done with FactoMineR, and then we used the function “fviz_pca_ind()” in factoextra package to plot the samples on the PCA map. To draw the dendrogram, we calculated the distances between cells by “maximum” and plot the graph using the function “fviz_dend()” in factoextra package.

### Comparison between results from SCAN-seq and SUPeR-seq

The genes detected in each stage were calculated as those expressed in at least 10% of the cells at that stage (Fig 3C). We used the Seurat package in R to find markers for samples at each stage/cell type. Then, we extracted unique markers for each embryonic stage in SCAN-seq and SUPeR-seq data, respectively. Then, the matrix of overlapped genes between the SCAN-seq and SUPeR-seq samples of each stage was generated to draw the Sankey diagram (S4B Fig).

### Detection of single nucleotide polymorphism (SNP)

We obtained about 155G whole geneome sequencing data of DBA/2C mouse generated by Nova seq of illumine platform and used fastp (v0.20.1) [35] to filter low-quality reads with default parameters. Then, the high-quality reads were aligned to mouse genome (Ensembl, GRCm38.90) with bwa mem (v0.7.17) [37]. After removing the duplicate reads, GATK4 HaplotypeCaller (v4.1.5.0) [38] was applied to call SNPs. The homozygous SNPs with depth ≥30 were remained as DBA/2C specific SNPs, which was 4,494,566 in total.

### Identification of maternal and paternal transcripts

Based on the mouse genome (Ensembl, GRCm38.90) and DBA/2C specific SNPs, we constructed the DBA/2C reference genome, REF_DBA_2C. Then, we aligned full-length reads to each mouse genome (Ensembl, GRCm38.90) and REF_DBA_2C using minimap 2(v.2.10) [31]. We retained reads covering at least 60% of the shortest transcripts of genes for parental typing in each single cell. The remaining cDNA reads were determined C57BL/6J or DBA/2C allelic under 2 conditions:(1) if the read contains only 1 strain-specific SNP, it is assigned to the corresponding strain; and (2) if the read had more than 1 strain-specific SNPs, it can be assigned to 1 strain only when the number of SNPs assigned to this strain is at least twice of that assigned to the other strain.

## Supporting information

S1 Fig Preprocessing of SCAN-seq data and quality control. (A) Schematic of SCAN-seq data pretreatments (for details, see Methods). (B) The length distribution of cDNA products before library construction. (C, D) Length distribution of full-length reads. The numerical data are listed in S2 Data. SCAN-seq, single cell amplification and sequencing of full-length RNAs by Nanopore platform.
(TIF)

S2 Fig Data quality of mESCs using SCAN-seq. (A) Saturation curve of detected genes and isoforms in mESC. (B) Heatmap of correlation value (Pearson) between each pair of mESCs. The correlation value of mESCs by SCAN-seq was even better than that by SUPeR-seq and Tang 2009 method. (C) Coverage of reads along the whole transcripts. (A–C) The numerical data are listed in S2 Data. mESC, mouse embryonic stem cell; SCAN-seq, single cell amplification and sequencing of full-length RNAs by Nanopore platform; SUPeR-seq, single-cell universal poly(A)-independent RNA sequencing.
(TIF)

S3 Fig Saturation curve of detected genes and isoforms in cells at different developmental stages. The numerical data are listed in S2 Data.
(TIF)

S4 Fig Gene expression analysis in mouse embryonic samples. (A) Number of detected genes in each individual cell at each developmental stage/type. The numerical data are listed in S2 Data. (B) Correspondence of stage-specific genes detected using SCAN-seq and SUPeR-seq. (C) GO analysis of the 6 group of genes in Fig 3D. GO, gene ontology; SCAN-seq, single cell amplification and sequencing of full-length RNAs by Nanopore platform; SUPeR-seq, single-cell universal poly(A)-independent RNA sequencing.
(TIF)

S5 Fig LncRNAs detected in mouse preimplantation embryos. (A) The reads ratio of mESCs and all blastomeres at different developmental stages. The center represents the mean, and the error bars represent the SEM. (B) Heatmap showing the expression levels of LncRNAs in all cells. (A, B) The numerical data are listed in S2 Data. lncRNA, long noncoding RNA; mESC, mouse embryonic stem cell; SEM, standard error of the mean.
(TIF)

S6 Fig Percentage of genes which can be assigned to each allele in each individual cell. The numerical data are listed in S2 Data.
(TIF)

S1 Table Developmental stage-specific genes in mouse preimplantation embryos. (XLSX)

S2 Table Unannotated transcripts identified in mouse oocytes and preimplantation embryo samples. (XLSX)

S3 Table Allele-specific transcripts identified under each mapping conditions. Each name of the column contains 3 parts of information: sample id, mapping reference, assigned strain.
(XLSX)

S4 Table LncRNAs detected in mouse oocytes and preimplantation embryos. lncRNA, long noncoding RNA.
(XLS)

S5 Table Summary of strand information of all full-length reads in each individual cell. (XLSX)

S1 Data The individual numerical values in Figs 1B and 1C, 2B, 3A, 3C, 3D, 4B–4D and 5A–5C. (XLSX)

S2 Data The individual numerical values in S1C, S1D, S2A–S2C, S3, S4, S5A, S5B and S6 Figs. (XLSX)

## Abbreviations

CJ: combination of known junctions
CS: combination of known splice sites
DBA: DBA/2NCrl
DEG: differentially expressed gene
ERCC: External RNA Controls Consortium
GO: gene ontology
HCA: human cell atlas
hCG: human chorionic gonadotropin
ICM: inner cell mass
IR: intron retention
LNA: locked nucleic acid
lncRNA: long noncoding RNA
mESC: mouse embryonic stem cell
MII: metaphase II
MIR: mono-exon by intron retention
NGS: next generation sequencing
nt: nucleotide
PCA: principal component analysis
PMSG: pregnant mare’s serum gonadotropin
RAGE-seq: Repertoire and Gene Expression by Sequencing
R2C2: Rolling Circle Amplification to Concatemeric Consensus
RT: reverse transcription
RT-PCR: reverse transcription PCR
SCAN-seq: single-cell amplification and sequencing of full-length RNAs by Nanopore platform
ScISOr-Seq: single-cell isoform RNA-seq
ScNaUmi-seq: Single-cell Nanopore sequencing with UMIs
scRNA-seq: single-cell RNA sequencing
SNP: single nucleotide polymorphism
SUPeR-seq: single-cell universal poly(A)-independent RNA sequencing
TE: trophectoderm
TGS: third-generation sequencing
UMI: unique molecular identifier
