# Supplementary figures and images for: Single-cell RNA-seq analysis of mouse preimplantation embryos by third-generation sequencing
Source: PLoS Biol. 2020 Dec 30;18(12):e3001017. doi: 10.1371/journal.pbio.3001017 (PMC7773192; doi:10.1371/journal.pbio.3001017)

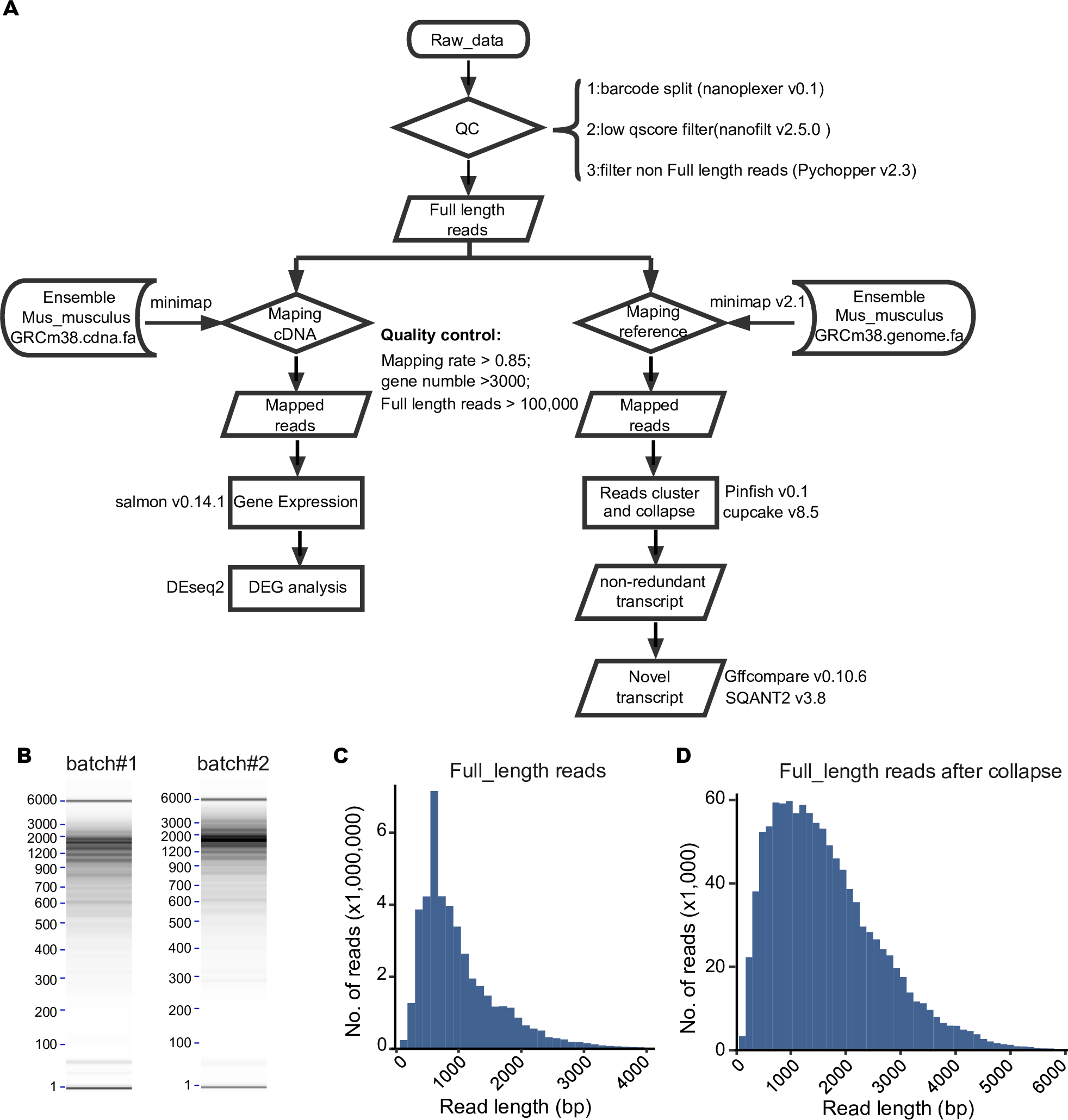

Supplement: S1 Fig — (A) Schematic of SCAN-seq data pretreatments (for details, see Methods). (B) The length distribution of cDNA products before library construction. (C, D) Length distribution of full-length reads. The numerical data are listed in S2 Data. SCAN-seq, single cell amplification and sequencing of full-length RNAs by Nanopore platform. (TIF) [file pbio.3001017.s001.tif]

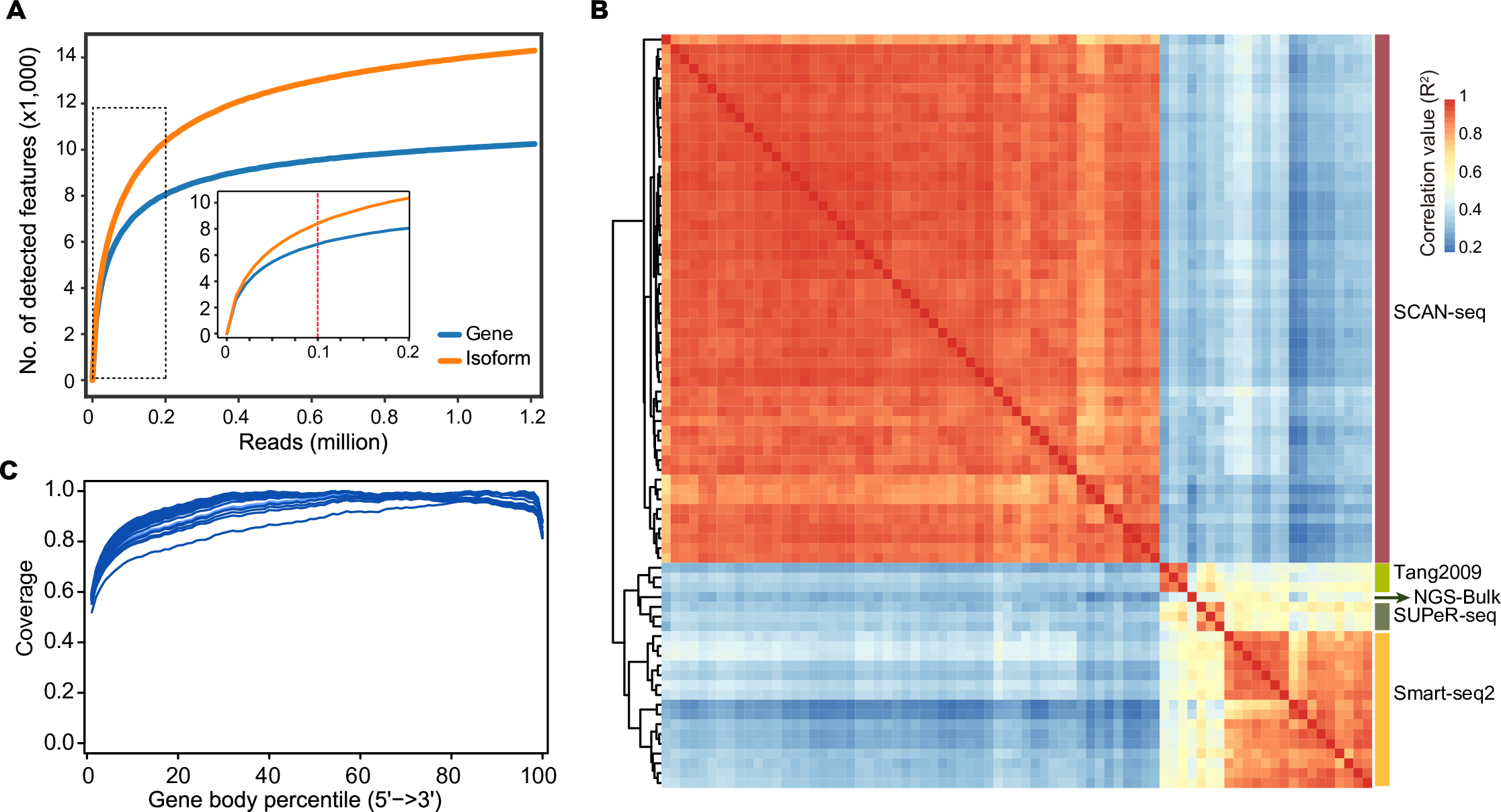

Supplement: S2 Fig — (A) Saturation curve of detected genes and isoforms in mESC. (B) Heatmap of correlation value (Pearson) between each pair of mESCs. The correlation value of mESCs by SCAN-seq was even better than that by SUPeR-seq and Tang 2009 method. (C) Coverage of reads along the whole transcripts. (A–C) The numerical data are listed in S2 Data. mESC, mouse embryonic stem cell; SCAN-seq, single cell amplification and sequencing of full-length RNAs by Nanopore platform; SUPeR-seq, single-cell universal poly(A)-independent RNA sequencing. (TIF) [file pbio.3001017.s002.tif]

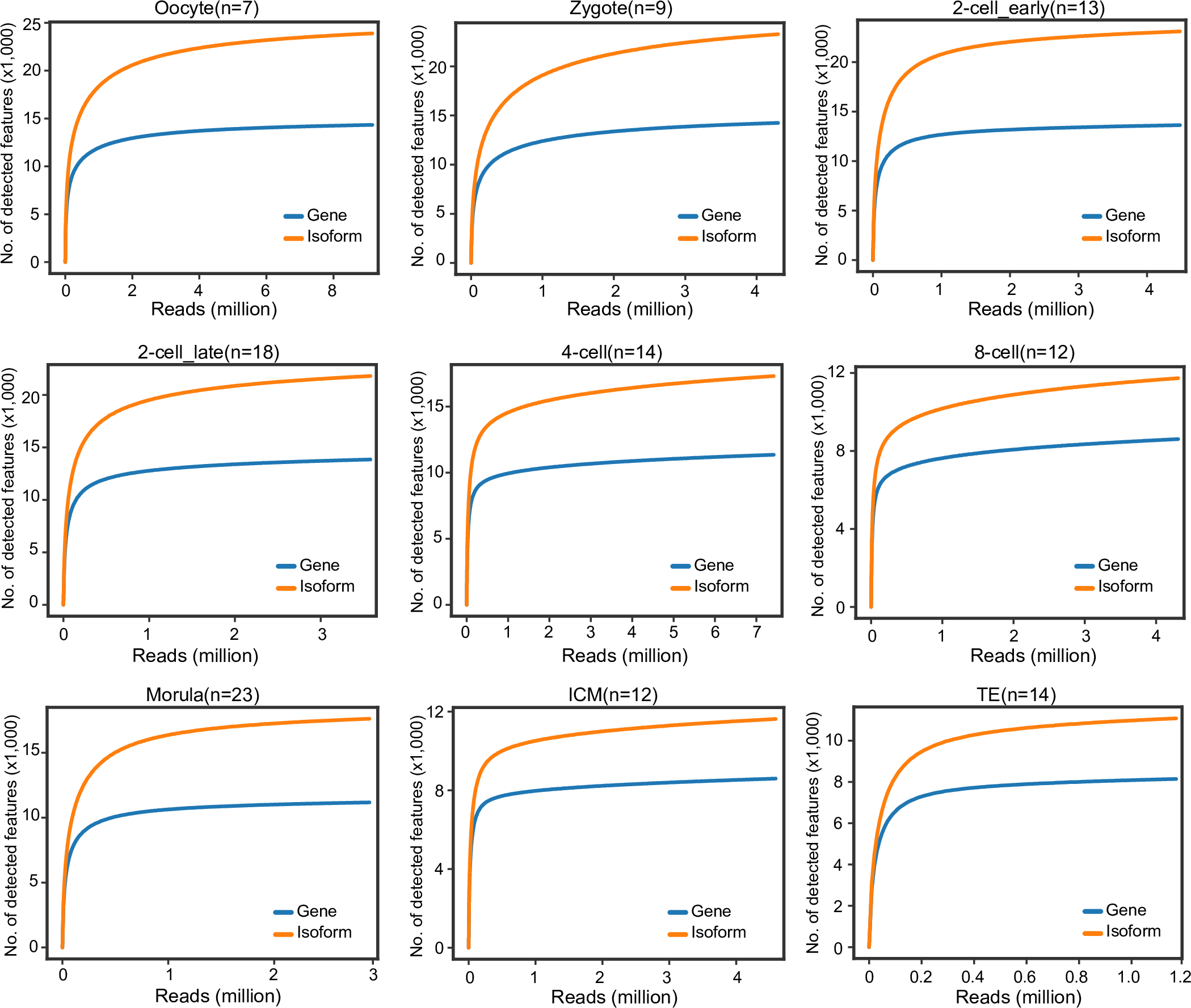

Supplement: S3 Fig — The numerical data are listed in S2 Data. (TIF) [file pbio.3001017.s003.tif]

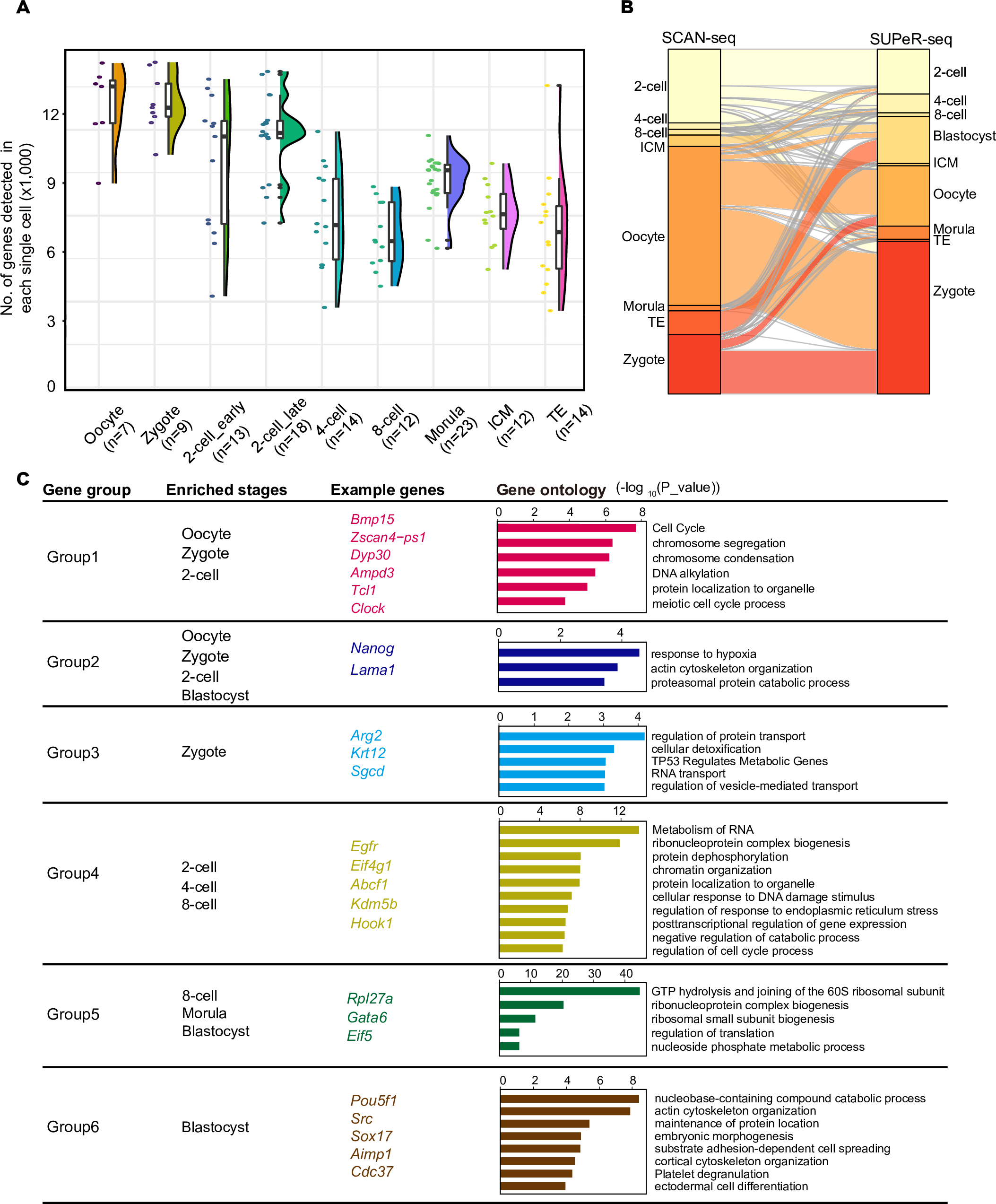

Supplement: S4 Fig — (A) Number of detected genes in each individual cell at each developmental stage/type. The numerical data are listed in S2 Data. (B) Correspondence of stage-specific genes detected using SCAN-seq and SUPeR-seq. (C) GO analysis of the 6 group of genes in Fig 3D. GO, gene ontology; SCAN-seq, single cell amplification and sequencing of full-length RNAs by Nanopore platform; SUPeR-seq, single-cell universal poly(A)-independent RNA sequencing. (TIF) [file pbio.3001017.s004.tif]

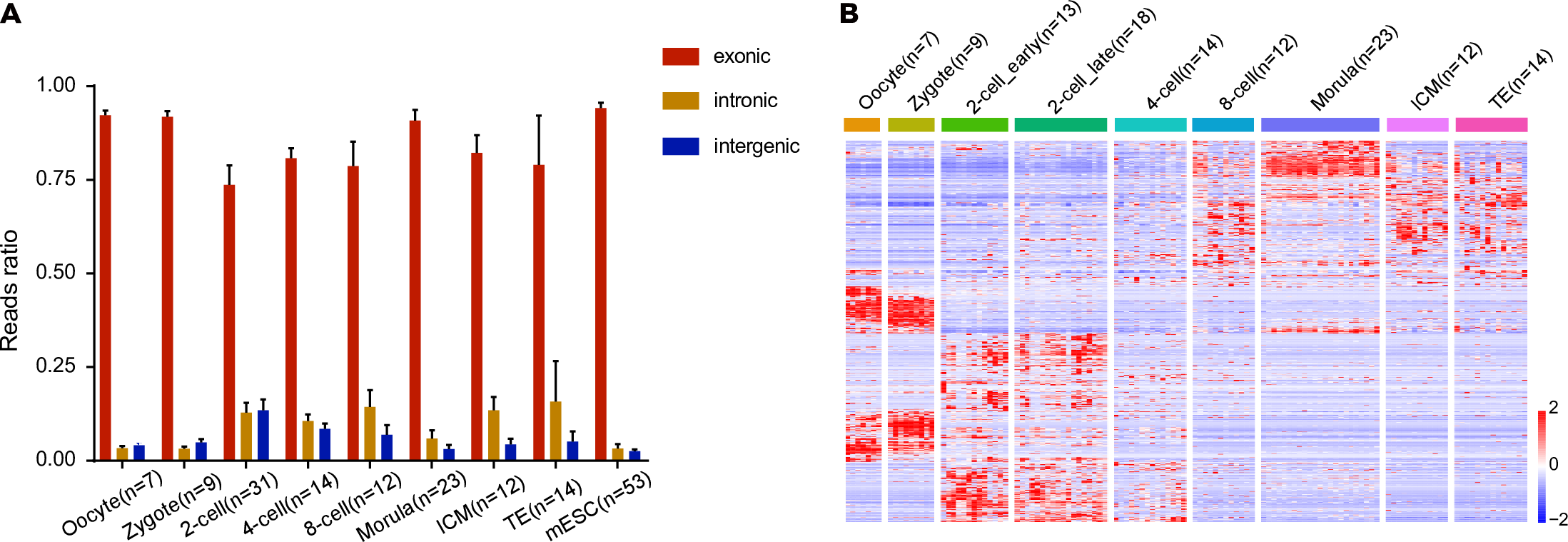

Supplement: S5 Fig — (A) The reads ratio of mESCs and all blastomeres at different developmental stages. The center represents the mean, and the error bars represent the SEM. (B) Heatmap showing the expression levels of LncRNAs in all cells. (A, B) The numerical data are listed in S2 Data. lncRNA, long noncoding RNA; mESC, mouse embryonic stem cell; SEM, standard error of the mean. (TIF) [file pbio.3001017.s005.tif]

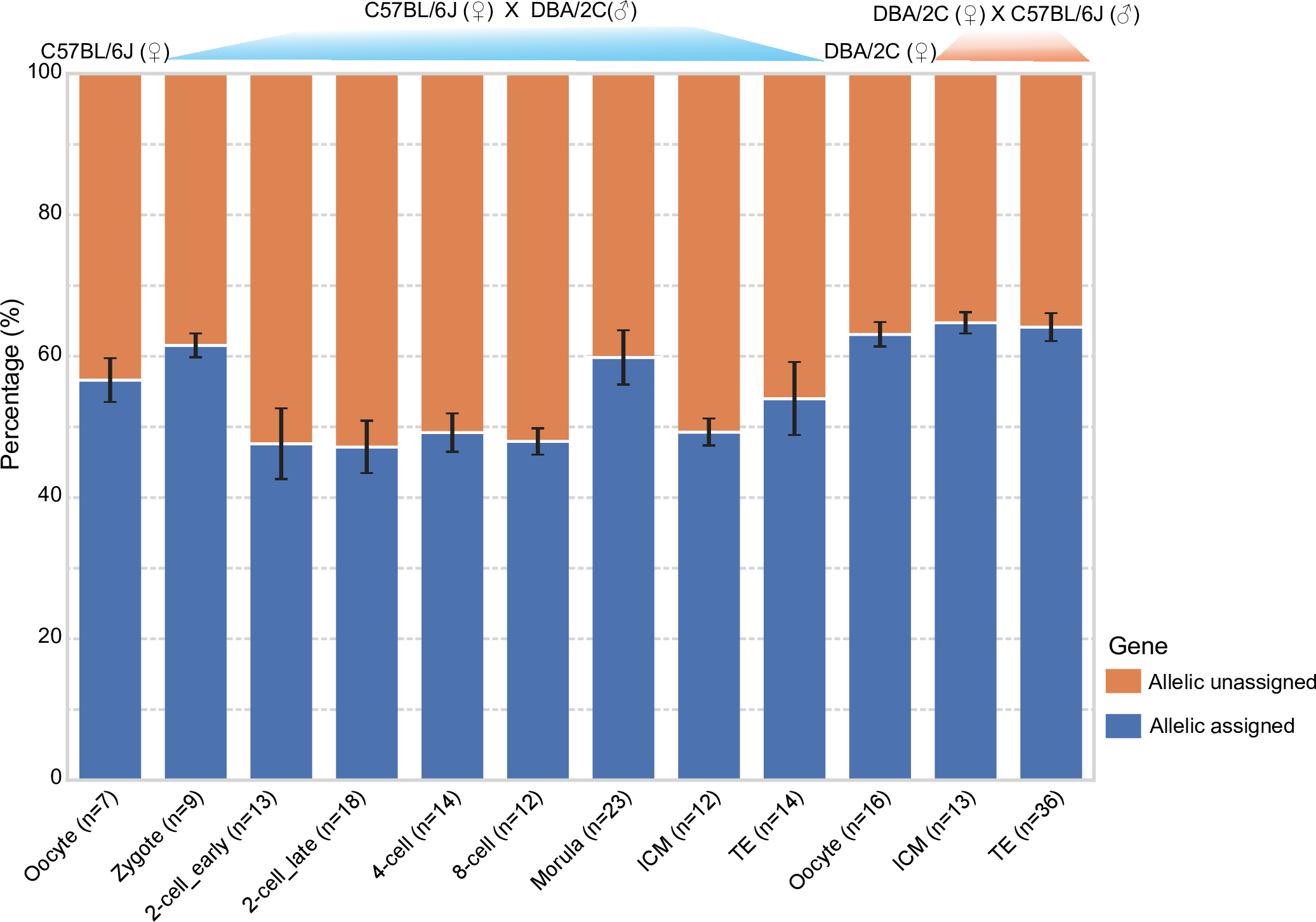

Supplement: S6 Fig — The numerical data are listed in S2 Data. (TIF) [file pbio.3001017.s006.tif]
